# Some Facts about Diphtheria

**Published:** 1882-03

**Authors:** 


### SOME FACTS ABOUT DIPHTHERIA.

The subject of diphtheria was opened by a paper from Dr. Jacobi, of New York. He said that the nature of the contagion was probably chemical. The presence of bacteria in diphtheria did not prove its parasitic character. The entrance of the diphtheritic poison was not the same in all cases. In some the origin of the disease was local. In others the poisoning of the blood by inhalation was the first step. In some, both modes of infection acted simultaneously. Diphtheria was very contagious. Both the patient and his surroundings, dwelling, furniture, towels, visitors, etc., conveyed the disease, and might do so after a long time. The contagion rose upward with the current of warm air in dwellings. It clung mostly to mucous membranes not endowed with many muciparous follicles. Mild cases might communicate serious ones, and *vice versa ;* and recent wounds were affected easily and speedily. The disease had been contracted from animals. It attacked children under three months of age, and those over seven or eight mostly, and mainly such as had been affected previously. Local paralysis of the soft palate, and sometimes of the muscles of deglutition and the glottis often attended the local deposits on these parts, with œdematous swelling of the same. The diphtheritic paralysis, properly so called, was an affection of apparent convalescence. The majority of cases occurred after mild attacks; sometimes after those with but little fever and a slow general course. It did not usually occur in cases complicated with albuminuria and nephritis. There was no symptom during the attack indicating future paralysis. Albuminuria was often found in diphtheria, but was mostly no grave symptom. It appeared to be the result of rapid elimination of the poison. Acute diffuse nephritis appeared at an earlier period than in scarlatina.—*Trans. Int. Med. Congress.*

